# Finite Element and Finite Volume Modelling of Friction Drilling HSLA Steel under Experimental Comparison

**DOI:** 10.3390/ma14205997

**Published:** 2021-10-12

**Authors:** Bernd-Arno Behrens, Klaus Dröder, André Hürkamp, Marcel Droß, Hendrik Wester, Eugen Stockburger

**Affiliations:** 1 Institute of Metal Forming and Forming Machines, Leibniz Universität Hannover, 30823 Garbsen, Germany; behrens@ifum.uni-hannover.de (B.-A.B.); wester@ifum.uni-hannover.de (H.W.); 2 Institute of Machine Tools and Production Technology, Technische Universität Braunschweig, 38106 Braunschweig, Germany; k.droeder@tu-braunschweig.de (K.D.); a.huerkamp@tu-braunschweig.de (A.H.); m.dross@tu-braunschweig.de (M.D.)

**Keywords:** tensile testing, material characterisation, material modelling, friction drilling, high-strength low-alloy steel

## Abstract

Friction drilling is a widely used process to produce bushings in sheet materials, which are processed further by thread forming to create a connection port. Previous studies focused on the process parameters and did not pay detailed attention to the material flow of the bushing. In order to describe the material behaviour during a friction drilling process realistically, a detailed material characterisation was carried out. Temperature, strain rate, and rolling direction dependent tensile tests were performed. The results were used to parametrise the Johnson–Cook hardening and failure model. With the material data, numerical models of the friction drilling were created using the finite element method in 3D as well as 2D, and the finite volume method in 3D. Furthermore, friction drilling tests were carried out and analysed. The experimental results were compared with the numerical findings to evaluate which modelling method could describe the friction drilling process best. Highest imaging quality to reality was shown by the finite volume method in comparison to the experiments regarding the material flow and the geometry of the bushing.

## 1. Introduction

Modern lightweight construction concepts for large-scale automotive production are increasingly characterised by the use of hybrid material composites of fibre-reinforced plastics (FRP) and metals. Resource-efficient lightweight construction aims to exploit material-specific potential through appropriate material combinations. Various processes are currently being used to produce hybrid material compounds, such as hybrid injection moulding. Industrially manufactured injection-moulded components use the metal insert as an element of load transfer from the metallic joining zone with other machine components into the plastic reinforcement. On some components, the bonding between the plastic and metal is realised with through-moulding points. One way to improve the low bond strength of these components is to insert friction-drilled bushings into the component before injection moulding. The interlocking effect between the plastic and the bushing can increase the load-bearing capacity of the hybrid material bonding in the component.

Previous studies mainly used the same modelling techniques and focused on the simulation of the friction drilling process parameters such as force or torque, temperature distribution, and development, as well as the validation of these values. So far, few publications have investigated the material flow and geometry of the bushing in, as well as against, the feed direction, since it is not necessary for common subsequent processes such as thread forming. Depending on the application, however, the correct digital mapping of the geometry is essential for a realistic computer-aided design of the process.

Therefore, this study focuses on proper digital mapping of the bushing formation while friction drilling. First, a material characterisation of the HX220 steel by means of temperature, strain rate, and rolling direction dependent tensile tests was performed. The parameters of the Johnson–Cook hardening and failure models were determined using the tensile tests. Second, experimental friction drilling tests of HX220 were conducted as well as analysed, and compared with friction drilling tests of HX420. Finally, the friction drilling tests of HX220 were modelled with different numerical approaches using the characterised material data and contrasted with the experiments. Investigated methods were the finite element (FE) in 2D and 3D. In addition, the finite volume (FV) method in 3D by means of the Coupled Eulerian–Lagrangian (CEL) method was applied and tested to model a friction drilling process.

### 1.1. Friction Drilling

Friction drilling is a noncutting manufacturing process for the production of bushings in sheet metal and hollow profiles. For this purpose, a rotating tungsten carbide friction drill is pressed onto the work piece under axial force. The frictional heat generated between the drill and the work piece reduces the strength of the material to such an extent that the drill can be pressed through the sheet metal with significantly reduced force. Material is shifted by the drill, and a bushing is formed outside of the sheet metal. The lower part of the bushing consists of approximately two-thirds of the displaced material volume. Upwards against the feed direction, the remaining third is displaced and can be formed into a collar [1]. In combination with thread forming, which is also chip-free, highly resistant threads can be formed in these bushings. Thus, the process offers ideal conditions for the production of detachable connections in thin-walled work pieces. In order to exploit further areas of application and to optimise the processes, computer-aided modelling is indispensable for time- and cost-saving reasons [2]. FE modelling allows the efficient investigation of most important process variables and their effect, especially for parameters that are not experimentally determinable [3].

### 1.2. Friction Drilling Simulalation

The heat transfer of a friction drilling process was modelled in 2D using the thermal FE method in Ansys by Miller et al. [4]. Obtained from experiment and simulation, the trend of temperature increase matched well. Abaqus/CAE(Simulia (Dassault Systèmes), Johnston, Rhode Island, US) was used by Miller et al. in [5] to model friction drilling of AA 6061-T6 in 3D with FE using eight-node hexagonal elements, adaptive meshing, and element deletion. The criterion to delete an element was based on the value of critical plastic strain. A threshold value of 2.2 was required to be reached before an element was deleted. A comparison of simulated and measured force, torque, and temperature showed a good quality of the model. Since the simulation showed no bushing against the feed direction, it was concluded that the element deletion removed too many elements. It was suggested to improve the simulation by developing a model without using the element deletion. The results in [6] by Krasauskas et al. also showed no bushing against the feed direction in a similar simulation model in Abaqus/CAE. Dehghan et al. used a 3D FE model to analyse the stress, plastic strain, and temperature distribution in friction drilling of AA 6061 [7]. The model was also created in Abaqus/CAE with hexahedral C3D8RT elements with a 0.1 mm element size, an adaptive mesh technique, and a Johnson–Cook failure model. The modelling showed reasonable results regarding the process parameters, but a comparison of the cross-section view of the experimental and simulated friction drilled hole showed a difference in both bushings. The simulation had a shorter bushing, most likely due to many elements deleted by the failure model. The heat generation and bushing formation caused by friction in friction drilling were studied by Dehghan et al. in [8]. A FE model in Abaqus/CAE was used, and examined the materials were AISI 304, Ti-6Al-4 V, and Inconel718. The model consisted of the Johnson–Cook failure model, and the damage evolution law was modified to include the Hillerborg’s fracture energy. The authors were able to represent the formation of the bushing well. Nevertheless, the bushing in the direction of feed was sometimes too short, and the bushing against the direction of feed was always too small.

Oezkaya et al. presented another 3D FE simulation model of a friction drilling in [9] for AlSi10Mg using the software DEFORM-3D, tetrahedral elements, and an adaptive mesh technique. A failure criterion was not mentioned. The experimental and numerical force, torque, and temperature showed a good agreement. A comparison of the bushing for experiment and simulation was not shown, but the simulation indicated the formation of a too-short bushing. The authors in [10,11,12] used a similar approach to model friction drilling processes: 3D FE models were created in DEFORM-3D and consisted of tetrahedral elements with an adaptive mesh technique. The study did not describe which failure models were implemented. Hynes et al. analysed friction drilling of copper Cu2C numerically and validated it through experiments with regard to force and torque [10]. The numerical temperature was not validated, and the bushing geometry was not analysed. Srilatha et al. analysed the aluminium AA 7075 numerically in terms of stress, strain, and temperature at different rotational speeds and feed rates, but did not validate the studies [11]. Bilgin et al. considered the AISI 1020 for friction drilling [12]. The numerically calculated temperature, torque, and force matched well with the experimental values for different rotational speeds. Unfortunately, no figures for the final geometry of the friction drilling simulation were presented, so the bushing could not be evaluated.

Other promising approaches to modelling friction drilling are mesh-free computational approaches. Those approaches are developed to deal with large deformation-induced material failure in destructive manufacturing. A common approach is the element-free Galerkin (EFG) method. To treat material failure more physically in destructive manufacturing processes, a genuine mesh-free method, the smoothed particle Galerkin (SPG) method, was developed more recently. Wu et al. simulated friction drilling of an AISI 304 steel with the SPG method in LS-DYNA [13]. The work piece was discretised by SPG particles with a nodal distance of 0.2 mm. The Johnson–Cook hardening model was applied to describe the flow behaviour of the work piece. However, the material failure was not described by the Johnson–Cook failure model. The failure process was modelled by the SPG bond failure mechanism. The numerical results showed a good match with experimental values regarding force and torque without deleting parts of the discretisation, as in the FE simulation [14].

## 2. Materials and Methods

### 2.1. Material Characterisation

**Figure 1:**
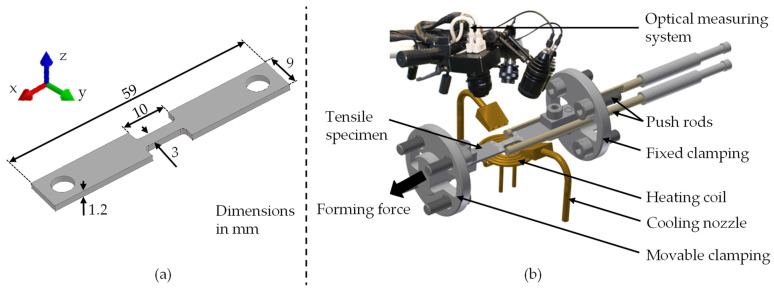
Miniature tensile test specimen geometry (**a**); and the experimental setup of the quenching and forming dilatometer with an optical measuring system (**b**).

To investigate the flow behaviour of the high-strength low-alloy (HSLA) HX220 steel, the miniaturised tensile test specimens shown in Figure 1a were used, as in [15]. The specimens were taken from a 1.2 mm thick sheet metal at 0°, 45°, and 90° to the rolling direction using water-jet cutting. Uniaxial tensile tests were carried out at elevated temperatures on a DIL 805A/D+T quenching(TA Instruments; New Castle; PA; USA) and forming dilatometer from TA Instruments. The test equipment of the forming dilatometer is shown in Figure 1b. Before testing, a stochastic pattern was applied, and a thermocouple was welded onto the specimen to regulate the temperature. It was then placed in the dilatometer, heated to the respective forming temperature via a heating coil with an integrated cooling system, and held for 20 s. Afterwards, the forming force was applied via the hydraulic system until failure of the specimen occurred. During the forming of the specimen, the change in length and width of the test area was measured with an ARAMIS (GOM GmbH, 38122 Braunschweig, Germany) optical measuring system from GOM GmbH. The forming temperature was investigated at 20 °C, 250 °C, and 500 °C, and the strain rate at 0.01 s^−1^, 0.1 s^−1^, and 1 s^−1^. Every parameter combination was performed five times to ensure statistical coverage. The occurring force and the measured length change were transferred into uniaxial flow curves according to the state of the art [16]. Further, temperature-dependent anisotropy coefficients were derived for the strain rate of 0.01 s^−1^ according to the common procedure [17].

### 2.2. Modelling of the Flow and Failure Behaviour

Since forming simulations often show plastic strains that are higher than the plastic strain of the uniaxial tensile test, extrapolation approaches are used to extrapolate flow curves up to the increased plastic strains. Furthermore, the approximation of the flow curve by an analytical approach is advantageous, since the FE calculation no longer requires time-consuming interpolation between the supporting points of the flow curve, but can be calculated efficiently using the analytical approach. A commonly used extrapolation approach is the Johnson–Cook hardening model, as shown in Equation (1) [18]:

(1) $$k_{f,J\text{-}C}=(A+B\;\varepsilon_{\mathrm{pl}}^{n})\;\times \;(1+C\;\times \;\ln\;{\dot{\varepsilon }}_{\mathrm{norm}})\;\times \;{\left(1-(T_{\mathrm{norm}}\right)}^{m})$$

(2) $${\dot{\varepsilon }}_{\mathrm{norm}}={\dot{\varepsilon }}_{\mathrm{pl}}/{\dot{\varepsilon }}_{\mathrm{pl},0}$$

**Figure 2:**
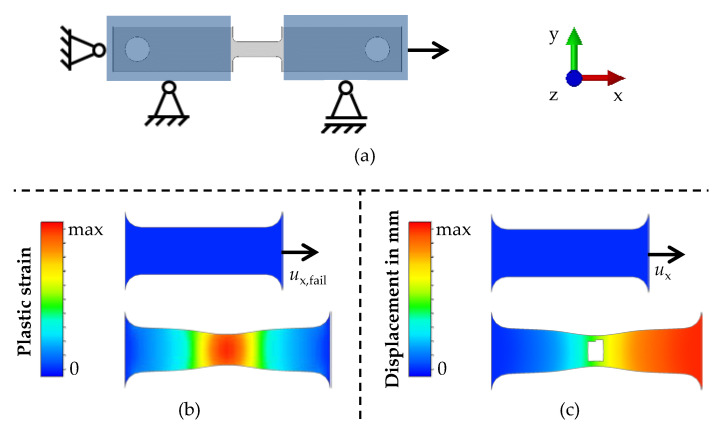
Geometry of the simulation model (**a**); and the boundary conditions for the comparison of the hardening model (**b**) and for the failure model (**c**).

*T*_norm_ = (*T* − *T*_room_)/(*T*_melt_ − *T*_room_) (3)

where *k*_f,J-C_ is the flow stress; *A*, *B*, *C*, *m*, and *n* are model parameters; $\varepsilon_{\mathrm{pl}}$ is the plastic strain; and ${\dot{\varepsilon }}_{\mathrm{norm}}$ and *T*_norm_ are the nondimensional plastic strain rate and the nondimensional temperature, respectively. ${\dot{\varepsilon }}_{\mathrm{norm}}$ is a function of the plastic strain rate ${\dot{\varepsilon }}_{\mathrm{pl}}$ and the reference plastic strain rate ${\dot{\varepsilon }}_{\mathrm{pl},\;0}$, while *T*_norm_ is a function of the temperature *T*, the room temperature *T*_room_, and the melting temperature of the material *T*_melt_. The model was parameterised using the experimental flow curves and the least squares method. For a comparison of the material flow behaviour and a first validation, the tensile tests were simulated. The 3D FE simulation model and the associated boundary conditions are shown in Figure 2a.

The geometry of the specimen, material data, and the boundary conditions were implemented in Simufact Forming 16.0 (simufact engineering gmbh, 21079 Hamburg, Germany) Only the middle area of the specimen, which was the plastic deformed area in the experiments, was modelled using hexahedral solid-shell elements with five integration points over the thickness and an element edge length of 0.1 mm. Adaptive remeshing was used when the plastic strain exceeded a value of 0.4. For the material properties, the Johnson–Cook hardening model and the anisotropy parameters by means of the Hill’48 yield criterion [19] were used. As boundary conditions, the left nodes were fixed in all directions, and the right nodes were freely movable only in the x-direction, in which a displacement was applied with the same speed as in the experiment. The end of the simulation was defined by the end of the displacement in the x-direction, which was the average value of five experiments for the displacement at failure *u*_x,fail_, as shown in Figure 2b. The problem was solved using an implicit solver. Finally, the force–displacement curves from the experiments and simulations were compared.

To predict the material failure in sheet metals, usually forming limit curves are used [20]. Due to the deformation during friction drilling, however, a stress state is present, which is not represented by forming limit curves. Therefore, the Johnson–Cook failure model [18] was used as depicted in Equation (4):

(4) $$\varepsilon_{f}=(D_{1}\;\times \;D_{2}\;\times \;\exp(-D_{3}\;\times \;\eta ))\;\times \;(1+D_{4}\;\times \;{\dot{\varepsilon }}_{\mathrm{norm}})\;\times \;(1+D_{5}\;\times \;T_{\mathrm{norm}})$$

where *ε*_f_ is the failure strain, *D*_1_ to *D*_5_ are model parameters, and *η* is the triaxiality. In the prior FE simulation of the tensile tests, the triaxiality and the plastic strain were evaluated at the location and time of the material failure of the specimen, which both were determined in the experiments. From the plastic strain–triaxiality curve, the area-weighted triaxiality was calculated, and the maximum plastic strain was defined as the failure strain. To calculate the Johnson–Cook failure model parameters, both the area-weighted triaxiality and the failure strain were used when applying the least squares method. For another comparison of the material failure behaviour, the tensile tests were simulated using the prior 3D FE models including material damage. The Johnson–Cook failure model and element deletion were implemented. Further, the boundary conditions were changed as shown in Figure 2c. The displacement in the x-direction *u*_x_ was set to a high value, since the end of the simulation was determined by deletion of the first element. Finally, the force–displacement curves from the experiments and simulations were compared to evaluate the moment of failure prediction by the simulation.

### 2.3. Friction Drilling Experiments

**Figure 3:**
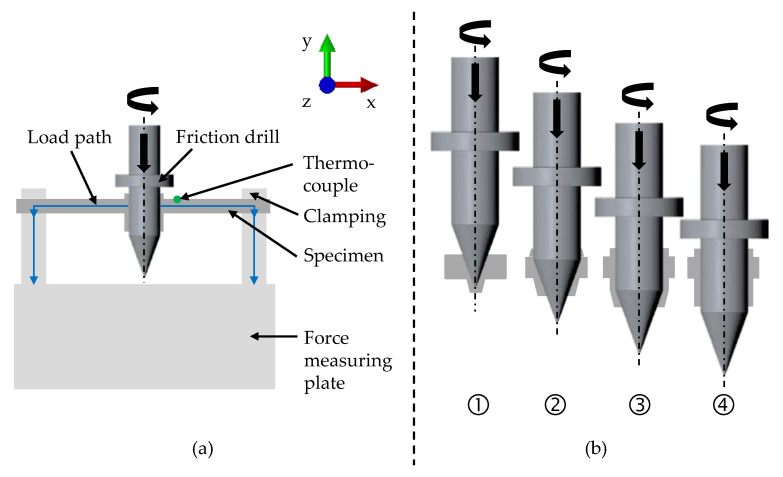
Schematic illustration of the experimental setup for friction drilling with force measurement (**a**) and states of the friction drilling (**b**): entering (**1**) and breakthrough (**2**) of the conical part of the friction drill; and entering (**3**) and breakthrough (**4**) of the cylindrical part.

For the experimental investigations, friction drilling tests were performed. The test setup was installed in a 5-axis DMU 100 Monoblock milling machine from DMG MORI(DMG MORI Aktiengesellschaft, 33689 Bielefeld, Germany), and consisted of a friction drill, a specimen with dimensions of 25 mm × 100 mm, and clamping and force-measuring plates. A schematic illustration of the experimental setup is shown in Figure 3a to clarify the resulting load path. For the experiments, a M4 friction drill and a separating agent from Flowdrill (Flowdrill Fließformwerkzeuge GmbH, 69469 Weinheim, Germany) were used. The tested rotational speed and feed rate were 5800 rpm and 50 mm/min. The resulting forming force while friction drilling was measured with the force-measuring plate. Measuring of the temperature on the bushing during the experiment was difficult due to the wide range of temperatures, the speed of the process, and the experimental setup. Therefore, thermocouples were welded on the specimens before testing to measure the temperature development. They were placed about 4 mm from the centre of the former. The tested specimens were made of the HX220 steel with a sheet thickness of 1.2 mm. To compare the influence of a higher sheet thickness and a higher material strength on the process, HX420 steel measuring 1.5 mm was also used in the friction drilling experiments. The forming was stopped at different strokes, such as 2 mm, 4 mm, 6 mm, and 8 mm. It was aimed to set the characteristic states entering (1) and breakthrough (2) of the conical part of the friction drill, as well as entering (3) and breakthrough (4) of the cylindrical part, as shown in Figure 3b. Therefore, it was possible to compare the material flow and resulting geometry of the bushing from the experiments to the simulation. Each experiment was performed five times to ensure statistical safety. Subsequently, the specimens were photographed from the outside with a VR-3200 3D profilometer from Keyence (KEYENCE DEUTSCHLAND GmbH, Neu-Isenburg, Germany). Further, a 3D profile of the upper and lower bushing was recorded with the 3D profilometer. Finally, the specimens were separated, mounted, grinded, and polished. The mounted separations also were microscopically examined with the 3D profilometer.

### 2.4. Numerical Modelling of Friction Drilling

Due to the very complex physical phenomena of the friction drilling process, several assumptions were made in the numerical models. The contour of the friction drill with its forming studs was simplified to be rotationally symmetrical. Furthermore, the friction during the friction drilling process was a complex and changing condition, and the friction parameters were difficult to determine experimentally. Therefore, the friction was simplified using the Coulomb friction law. To create the numerical models, the effective part of the tools and the specimen were abstracted, prepared, and discretised with a finite element mesh. The boundary conditions were chosen according to real friction drilling tests. Therefore, an M4 friction drill with a rotational speed of 5800 rpm and feed rate of 50 mm/min was simulated. The contact between the specimen and the clamp tool was modelled as adhesive, and for friction between the specimen and the friction drill, a Coulomb friction coefficient of 0.3 was assumed. The characterised and modelled material behaviour of the specimen was used by implementing the Hill’48 yield criterion, the Johnson–Cook hardening model, and the failure model.

Lagrangian discretisation is a meshing method with a matter-fixed arrangement [21]. The model sections are divided as finite elements of the component. A movement is represented in the matter-fixed elements as a movement of those elements. Each element is a part of the model and moves with the model. Limitations of the range of motion are represented as contact surfaces, which may not be penetrated by the elements. Lagrangian discretisation is common for the FE method, and hence for applications such as solid-state computations and structural mechanics [22].

**Figure 4:**
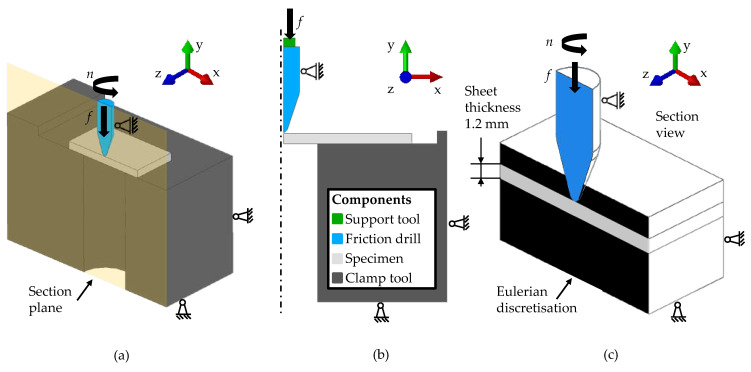
Geometry and boundary conditions of the numerical friction drilling models: section view of the 3D FE model (**a**); 2D FE model (**b**); and section view of the 3D FV model (**c**) with rotational speed *n* as well as feed rate *f*.

First in this study, a symmetrical 3D FE model of the friction drilling experiments was generated with the software Simufact Forming 16.0. Such 3D FE simulations are commonly used to model forming processes due to their accurate representation of reality [23]. With the high reality fidelity, the computation time increased massively. Figure 4a shows the geometry and the boundary conditions of the 3D FE model. The geometry consisted of the friction drill, the specimen, and a clamp tool. In preliminary simulations, the full geometry and different sections in 3D were investigated using axis symmetry to reduce the number of elements as much as possible. Finally, one-eighth of the geometry was modelled, because this offered the best ratio in terms of calculation time and result accuracy. The specimen was modelled as elastic-plastic, and the other parts as rigid bodies. Solid elements of the element type hexahedron with remeshing at plastic strains of 0.2 were used. The element edge length was 0.1 mm in the area of the bushing and 0.4 mm outside, also to reduce the computation time. In preliminary investigations, it was found that a coarser mesh in the area of the bushing led to a poorer reproduction of the friction drilling. An implicit scheme and an iterative solver were chosen for the computation.

Second, a rotationally symmetrical 2D FE model of the friction drilling experiments was created with the software Simufact Forming 16.0. Using symmetry had the advantage of reducing the number of elements and shortening computation time, and is therefore also often used in the literature [24]. Hence, it was useful to study the suitability of the 2D FE simulation to reproduce friction drilling numerically. The geometry and the boundary conditions of the 2D FE model are depicted in Figure 4b. A further support tool was added in the simulation. The specimen was modelled as elastic-plastic, the clamp tool and the support tool as rigid, and the friction drill as an elastic deformable body. A quad element with an element edge length of 0.1 mm was used to discretise the specimen and the friction drill. The remesh criterion for the specimen was set to a plastic strain of 0.2. As in the 3D FE simulation, implicit calculation and an iterative solver were used. The friction welding module of Simufact Forming 16.0 was used to model the friction drilling. Due to the friction between the rotating bodies in friction welding processes, the joining zone is heated locally and the flow stress is reduced. Similar processes take place in friction drilling processes, and therefore the module was applied. In the friction welding module, the frictional heat was not calculated via the tool movement, but via a substitute model [25]. This allowed simulating such processes as 2D, since rotations are not describable in 2D. The substitute model described the generation of frictional heat *q*_FR_ as a function of conducted frictional work *W*_FR_ and its dissipation coefficient *β*_FR_, as shown in Equation (5):

d*q*_FR(*i*)_ = *β*_FR_ × d*W*_FR(*i*)_ = *β*_FR_ × *τ*_R(i)_ × *ω* × *r* × d*t* (5)

The incremental frictional work of a node *i* on the contacting surface can be described as a function of its axial distance from the rotation centre *r*, current frictional shear stress *τ*_R(i)_, rotational speed *ω*, and incremental time d*t*.

Eulerian discretisation is a meshing method with a space-fixed arrangement [21]. The model sections are divided as a fixed network of elements in space. A movement is represented in the space-fixed elements as a balance of the quantity flowing in, the quantity flowing out and the quantity remaining in the elements. Limitations of the range of motion are represented as blocking of elements. A common method in Eulerian discretisation is the FV method, and therefore is used for applications such as fluid dynamics and flow processes. The CEL method has the possibility to couple an Eulerian formulation with a Lagrangian formulation to allow Eulerian and Lagrangian discretisation to interact within one model. The Lagrangian part can move through the Eulerian discretisation until it encounters an Eulerian material. Then, the contact between the different discretisations is calculated by a penalty contact method.

Third, in Abaqus/CAE, a 3D FV friction drilling model with CEL was built up. The geometry and the boundary conditions of the 3D FV model are illustrated in Figure 4c. It consisted of the friction drill, the specimen, and the Eulerian discretisation of the space around the specimen. The friction drill was modelled as elastic, and the specimen as elastic-plastic. For the Eulerian discretisation, which had a volume of 15 mm × 15 mm × 8 mm, solid elements of the element type hexahedron with an element edge length of 0.1 mm were used. The friction drill was discretised with solid tetrahedral elements with an element edge length of 0.5 mm and with a refinement of the mesh at the tip of the tool of 0.3 mm. A direct solver and explicit scheme were used to solve the model.

## 3. Results

### 3.1. Material Data

In Table 1, the chemical compositions of the investigated HX220 steel are given, as measured by spark spectroscopy. The determined chemical compositions were within the normed range specified by DIN EN 10268 [26].

**Figure 5:**
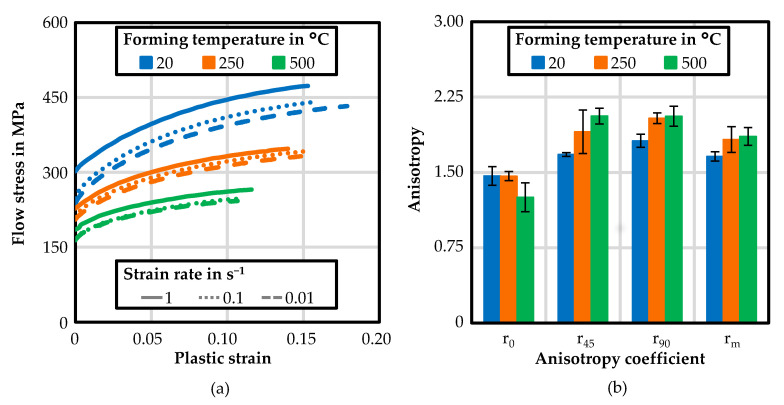
Experimental temperature and strain-ate- dependent flow curves at 0° to the rolling direction (**a**); and temperature-dependent anisotropy coefficients (**b**) for HX220 at a 1.2 mm sheet thickness.

The experimental flow curves of HX220 in 0° to the rolling direction are shown for plastic strain up to 0.2 in Figure 5a. In each case, it was the middle curve of the five recorded curves. As expected, the flow stress decreased with increasing forming temperature. With respect to strain rate dependence, no strong influence could be observed for the particular temperatures. However, the yield stress increased slightly with a higher strain rate. Temperature-dependent anisotropy coefficients with the standard deviation of the HX220 are depicted in Figure 5b. For all three investigated forming temperatures, the anisotropy values at 0°, 45°, and 90° to the rolling direction, as well as the mean perpendicular anisotropy coefficient, showed values significantly higher than 1. Therefore, anisotropic plastic behaviour was present, in which more material flowed from the width to the length than from the thickness under tensile load in the longitudinal direction. In this case, the sheet metal exhibited greater resistance to a reduction in sheet thickness.

**Figure 6:**
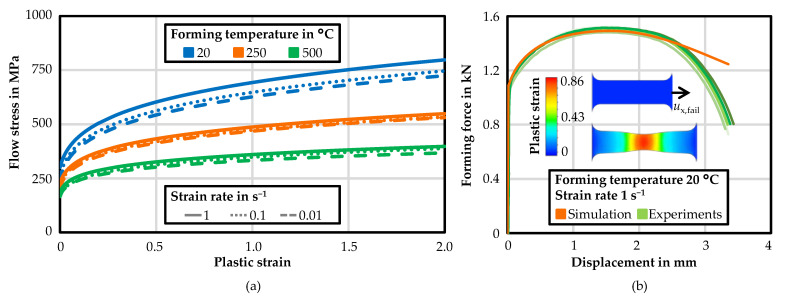
Extrapolated flow curves of HX220 for a plastic strain up to two (**a**); and comparison of the forming force–displacement curves from the experiments and simulation with average displacement at failure *u*_x,fail_ (**b**).

The experimentally recorded flow curves were approximated and extrapolated using the Johnson–Cook hardening model. The resulting flow curves are shown in Figure 6a for a plastic strain up to two. The identified parameters of the Johnson–Cook hardening model are listed in Table 2. For a comparison of the material modelling, the tensile tests were simulated using the Johnson–Cook hardening model and Hill’48 yield criterion. Figure 6b shows a comparison of the forming force-displacement function of five experiments, and the numerical simulation exemplarily for a forming temperature of 20 °C and a strain rate of 1 s^−1^. A very good agreement between the experimental results and the simulation was achieved. After the maximum forming force was reached, the simulated forming force decreased less strongly than in the experiments. This was because the calculation of the forming force in the simulation software did not take the damage evolution into account, which took place after the maximum forming force was reached and the necking occurred. Therefore, it was reasonable for the simulation to overestimate the experimental forces after the onset of necking.

**Figure 7:**
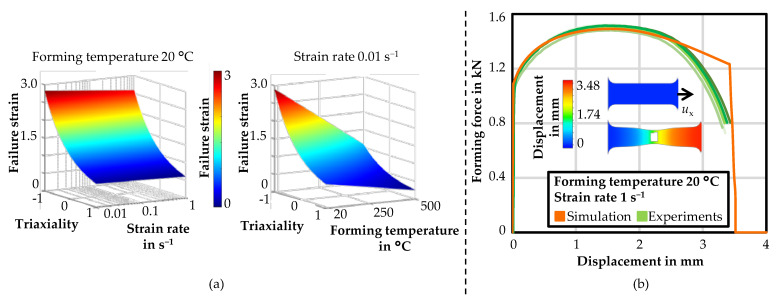
A 3D representation of the Johnson–Cook failure model for HX220 (**a**); and a comparison of the forming force–displacement curves from the experiments and simulation with displacement in x-direction *u*_x_ (**b**).

The Johnson–Cook failure model was parameterised based on the simulations of the tensile tests. A three-dimensional representation of the parameterised Johnson–Cook failure model is shown in Figure 7a for a forming temperature of 20 °C varying the strain rate, and for a strain rate of 0.01 s^−1^ varying the forming temperature.

The failure strain decreased marginally with an increase of the strain rate from 0.01 s^−1^ to 1 s^−1^. Increasing the forming temperature resulted in a large reduction of the failure strain. The calculated parameters are listed in Table 3 for HX220. For another comparison of the material modelling, the tensile tests were then simulated using the Johnson–Cook hardening and failure model. A comparison of the forming force-displacement function of five experiments and the numerical simulation exemplarily for a forming temperature of 20 °C and a strain rate of 1 s^−1^ is depicted in Figure 7b. The drop of the forming force in the simulation due to the deletion of the elements was in the range of the experiments. Overall, the simulation showed a good agreement with the failure model. Since the modelling of the material behaviour with the Johnson–Cook hardening and failure models matched well, the modelling approaches were used in the further simulations of the friction drilling process.

### 3.2. Experimental Results of Friction Drilling

**Figure 8:**
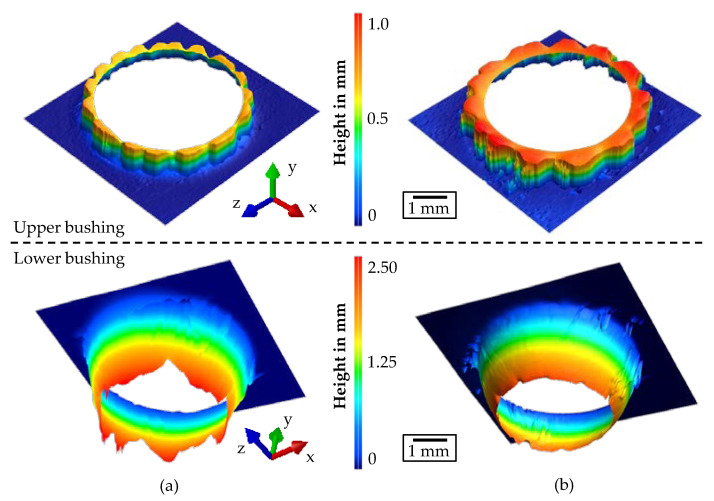
Optically measured 3D profiles of the upper and lower bushings for HX220 at a 1.2 mm sheet thickness (**a**) and HX420 at 1.5 mm (**b**).

Figure 8 illustrates the resulting bushings for HX220 and HX420 as optically measured 3D profiles. The upper bushing against the feed direction had a maximum length averaging of 0.72 mm for HX220 and 0.97 mm for HX420. The higher length of HX420 can be related to the higher sheet thickness of 1.5 mm compared to the thickness of 1.2 mm for HX220. A further cause of the higher length of HX420 was the higher tensile strength of 443 MPa in comparison to the tensile strength of 365 MPa for HX220. The gearlike structure of the upper bushings was due to the fact that the image was taken from above, and the small cracks in the upper bushing were projected onto the entire geometry. Lower bushings in the feed direction showed an inverse correlation. The maximum length for HX220 was on average 2.42 mm, and for HX420 slightly less than 2.38 mm. Cracks that appeared in the lower bushing of HX220 and not in HX420 can explain the cause of the almost equal lengths. Cracks indicated that the thickness of the lower bushing of HX220 was most likely reduced compared to HX420, and therefore increased the length of the lower bushing of HX220.

**Figure 9:**
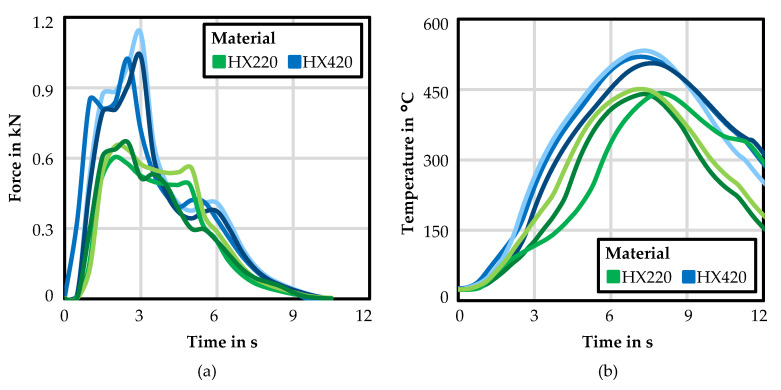
Experimental measured force–time curves (**a**) and temperature–time curves (**b**) for friction drilling HX220 at a 1.2 mm sheet thickness, as well as HX420 at 1.5 mm, for three repetitions each.

Experimental force–time curves are shown in Figure 9a for HX220 and HX420, for three measurements each. The maximum force of HX220 was on average 0.71 kN, and for HX420 was 1.12 kN. Higher forces while friction drilling HX420 compared to HX220 were due to the higher sheet thickness and the higher tensile strength of the HX420. In addition to the higher maximum, the force–time curves of both materials showed the same progression. The force rose fast after contact of the drill with the specimen, then rose until the maximum force during forming and decreased quickly after the material was perforated. Figure 9b depicts the experimental temperature–time curves for HX220 and HX420 placed at 2 mm from the resulting bushing for three measurements. The diagram clearly shows that the temperature measurements were reproducible. A similar progression for both materials was visible. The maximum temperature was higher for HX420 than for HX220 due to the higher material strength of HX420, and due to the higher plastic work. In addition, the higher sheet thickness of HX420 resulted in a higher maximum temperature due to the increased contact area, since more material was in contact with the drill. The average maximum temperature for HX220 was 444.2 °C, and for HX420 was 519.2 °C. It was also noticeable that the temperature rose almost as fast for HX220, but fell faster than for HX420 due to the smaller sheet thickness.

### 3.3. Numerical Results and Comparison to the Experiments

**Figure 10:**
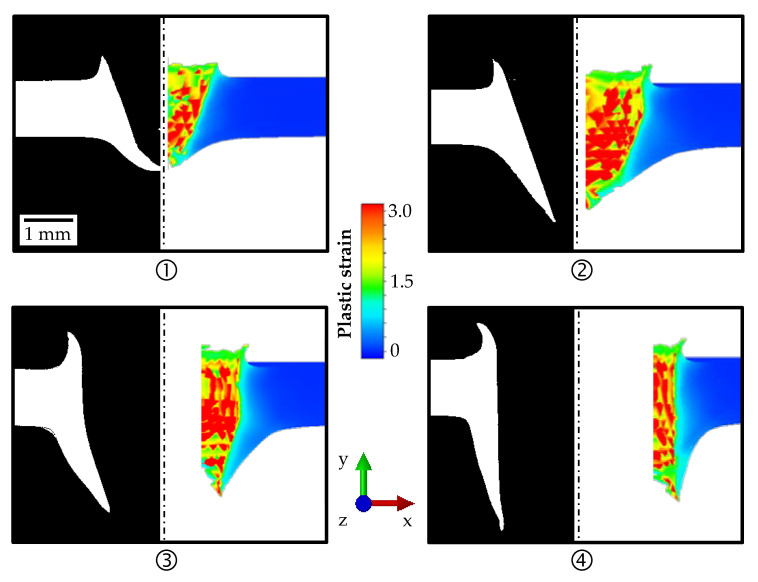
Comparison of the bushings for the experimental micrographs of the mounted separations and the 3D FE simulation at the states: entering (**1**) and breakthrough (**2**) of the conical part of the friction drill; and entering (**3**) and breakthrough (**4**) of the cylindrical part for HX220 at a 1.2 mm sheet thickness.

Figure 10 shows the results of the 3D FE simulation of a one-eighth geometry compared to the micrographs of the mounted separations for different strokes. Overall, the agreement between experiment and simulation was good for the different strokes. However, it was problematic that with increasing stroke, the plastic strain reached high values, the damage was accumulated, and thus many elements were removed. An even finer mesh in the area of the bushing did not improve the outcome and was computationally almost not practicable. For applications in which the upper and lower bushing were negligible, the simulation could be performed in 3D with FE. However, since the form of the bushing represented an important result variable in this study, further investigations in 2D with FE and in 3D with FV were carried out.

**Figure 11:**
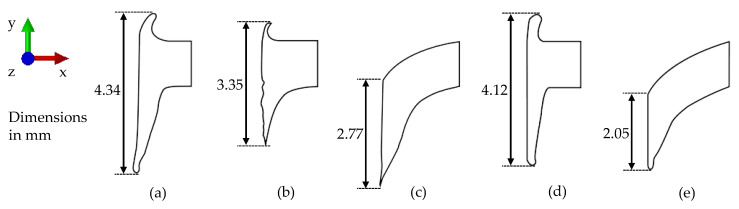
Geometry shapes of the resulting bushing for HX220 at a 1.2 mm sheet thickness: experiment (**a**); 3D FE (**b**); 2D FE (**c**); 3D FV (**d**); and 3D FV without rotation (**e**).

The results of all performed simulations and of the corresponding experiment are shown in Figure 11 as a comparison of the resulting geometry shapes of the bushing. Section views of the bushings were derived and measured digitally. The length of the bushing was measured from the upper bushing or from the upper side of the sheet to the lower bushing. While the diameter of the bushing was very similar for all simulations, the length of the upper and lower bushing differed greatly for the different simulation methods. Figure 11a,b depict the geometry shape from the experiment and the 3D FE simulation, as shown in Figure 10. The length of the bushing was 4.34 mm for the experiment and 3.35 mm for the 3D FE simulation. The rotationally symmetrical 2D FE model using the friction welding module from Simufact Forming 16.0 is shown in Figure 11c. It is clearly visible that no upper bushing was created while friction drilling, and the specimen was bent strongly, resulting in a shorter bushing length of 2.77 mm. For the numerical modelling of friction drilling, both the rotational and translational motion of the friction drill had to be modelled, and therefore a 3D FE simulation was necessary. Compared to rotational friction welding, the rotation also was required to be considered, and could not be simplified in a 2D FE simulation via an analytical approach for heat calculation. The 3D FV simulation in Figure 11d had a bushing with a 4.12 mm length, and was therefore the closest to the experiment. In addition, the thickness of the bushing was much more realistic than in the FE simulations. To prove the theory that both the rotational and translational motion of the friction drill had to be modelled, the 3D FV simulation was performed without the rotation boundary condition of the friction drill. The resulting bushing is illustrated in Figure 11e, and revealed similarities to the 2D FE simulation. The bushing for the 3D FV simulation without rotation was 2.05 mm.

**Figure 12:**
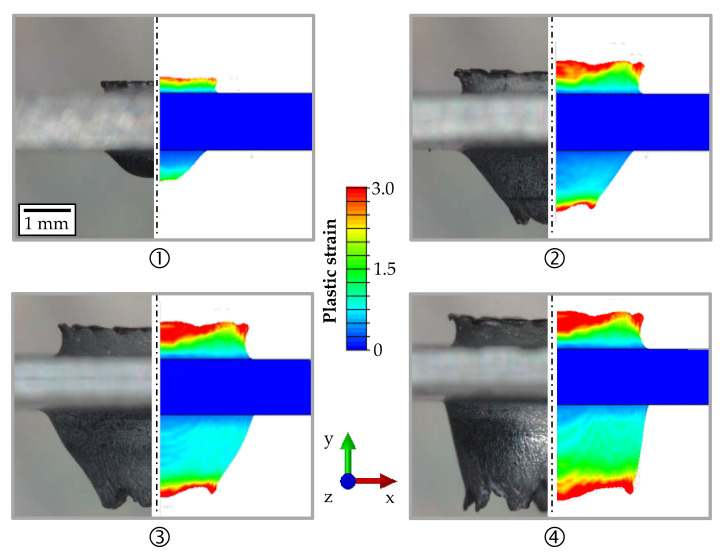
Comparison of the bushings for the experimental micrographs and the 3D FV simulation in the states: entering (**1**) and breakthrough (**2**) of the conical part of the friction drill; and entering (**3**) and breakthrough (**4**) of the cylindrical part for HX220 at a 1.2 mm sheet thickness.

Figure 12 illustrates the results of the 3D FV simulation compared to the experimental micrographs for the different strokes of 2 mm, 4 mm, 6 mm, and 8 mm. In Abaqus/CAE, it was not possible to generate a section view of the contour plot for the 3D FV simulation. Therefore, the plastic strain is shown as seen from the outside. In order to ensure a better comparison with the experiment, micrographs of the specimens from outside were taken in addition to the micrographs of the mounted separations. The numerical results fit very well to the experimental investigations regarding the form of the upper and lower bushing at each stroke. High plastic strains were also achieved in the simulation with FV, but significantly fewer elements were removed compared to the 3D FE simulation, resulting in a better geometric approximation of the bushing.

**Figure 13:**
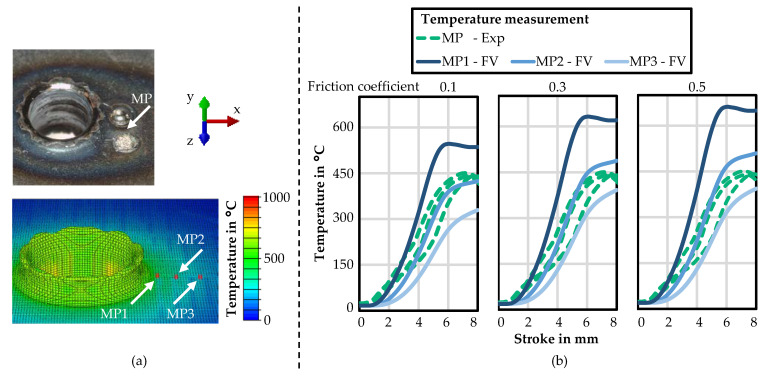
Temperature-measuring position in the experiments and in the FV simulation (**a**); and experimental measured and simulated temperature–stroke curves (**b**) for friction drilling HX220.

A comparison of the temperature measurements from the experiments and the FV simulation are shown in Figure 13. In Figure 13a, the position of the spot-welded thermocouple (MP) and three measuring points (MP1–3) are depicted. The thermocouple was placed about 2 mm from the bushing’s outer edge (i.e., approximately 4 mm from the centre of the former). In the simulation, the measuring points were positioned about 1 mm, 2 mm, and 3 mm from the bushing’s outer edge (i.e., approximately 3 mm, 4 mm, and 5 mm from the centre of the former). The measurements did not ensure a full validation of the simulation, but a good estimation and verification was possible. In Figure 13b, the corresponding temperature–stroke curves from three experiments and from the FV simulation at the three measuring points are shown. In addition to the previous simulation with a friction coefficient of 0.3, the simulation was carried out with friction coefficients of 0.1 and 0.5 to study the influence of the coefficient. For the three friction coefficients, the development of the temperature in the simulation was similar. As expected, the maximum temperature increased with an increasing friction coefficient. The MP2 in the simulations with friction coefficients 0.1 and 0.3 agreed well with the experiments, whereby the temperature was slightly overestimated at 0.3 and underestimated at 0.1.

## 4. Discussion

Equivalent to the state of the art, the 3D FE simulation showed restriction in reproducing the experimental length of the bushing. In particular, the upper bushing was very short. For applications in which the geometry of the bushing is negligible, the simulation can be performed in 3D with FE. To improve the 3D FE simulation, a new model to split the mesh, such as node separation method, would also be helpful. Using 2D FE methods usually seems to be a time- and cost-saving option that still maintains good model quality. Nevertheless, it was not possible to model friction drilling in 2D with an adequate model quality compared to the other methods in this research. In addition to the analytical approach for generating the temperature, an analytical approach for calculating the rotation and its effects would be necessary for the 2D simulation. The bushing formation could be mapped well using the FV simulation in 3D and more realistic compared to the other methods. It also offers optimisation potential in terms of the bushing geometry. The development of cracks in the bushing could not be reproduced realistically with the current model.

## 5. Conclusions

This paper presented experimental und numerical investigations of friction drilling HSLA steel. Temperature, strain rate, and rolling direction dependent tensile tests of the HSLA HX220 were executed and used to parametrise the Johnson–Cook hardening and failure models. Friction drilling tests were performed and analysed for HX220 and for HX420. Further, the experiments of HX220 were numerically modelled using different methods. First, the process was modelled using FE in 3D, similar to most of the literature. Then, a 2D FE model was created using a welding module. Last, the FV method was used to reproduce and analyse the friction drilling process numerically.

Since the comparison of the simulations and the experiments showed good agreement, it could be assumed that the methods used for the material characterisation and modelling were appropriate. Based on the findings, we concluded that a more realistic material flow and geometry of the bushing was achieved by the FV simulation, rather than the 3D and 2D FE simulation. It is important to note that the integration of the rotation in the simulation was important for the correct mapping of the bushing geometry.

With the help of the improved modelling of the bushing geometry, it was possible to carry out numerical investigations regarding the bushing as an interlocking element for injection moulding in further work. An overmoulded bushing under load could be numerically mapped and compared with experiments so that further digital investigations can be carried out with verification. An investigation of the arrangement of bushings as a pattern and the effect on the bond strength would be of interest. Once a suitable bushing arrangement has been identified, the concept can be transferred to a simulated component and validated.

## Figures and Tables

**Table 1:** Chemical composition of HX220 in mass %.

C	Si	Mn	P	S
0.0357	0.0305	0.4350	0.0366	0.0069

**Table 2:** Parameters of the Johnson–Cook hardening model for HX220.

*A* in MPa	*B* in MPa	*C*	*m*	*n*	${\dot{\varepsilon }}_{pl,0}$ in s^−1^	*T*_room_ in °C	*T*_melt_ in °C
212.2	409.2	0	0.7521	0.3524	0.01	20	1500

**Table 3:** Parameters of the Johnson–Cook failure model for HX220.

*D*_1_	*D*_2_	*D*_3_	*D*_4_	*D*_5_
0.3846	0.8279	−1.0827	−0.0189	−1.9109

## Data Availability

The data presented in this study are available upon request from the corresponding author. The data are not publicly available due to industrial confidentiality.
